# The Three-Step Approach for Lumbar Disk Herniation with Anatomical Insights Tailored for the Next Generation of Young Spine Surgeons

**DOI:** 10.3390/jcm13123571

**Published:** 2024-06-18

**Authors:** Giuseppe La Rocca, Gianluca Galieri, Edoardo Mazzucchi, Fabrizio Pignotti, Vittorio Orlando, Simona Pappalardo, Alessandro Olivi, Giovanni Sabatino

**Affiliations:** 1 Institute of Neurosurgery, Fondazione Policlinico Universitario A. Gemelli IRCCS, Catholic University, 00168 Rome, Italy; giuseppe.larocca@policlinicogemelli.it (G.L.R.); orlandovittorio.md@gmail.com (V.O.); alessandro.olivi@policlinicogemelli.it (A.O.); giovanni.sabatino@policlinicogemelli.it (G.S.); 2 Neurosurgical Training Center and Brain Research, Mater Olbia Hospital, 07026 Olbia, Italy; edoardo.mazzucchi@gmail.com (E.M.); fabrizio.pignotti@materolbia.com (F.P.); 3 Department of Neurosurgery, IRCCS Regina Elena National Cancer Institute, 00144 Rome, Italy; 4 Department of Neurosurgery, Mater Olbia Hospital, 07026 Olbia, Italy; 5 Department of Anatomical Pathology, Giovanni Paolo II Hospital, 97100 Olbia, Italy; simonapappy@gmail.com

**Keywords:** lumbar disk herniation, three-step approach, surgical triangle, discectomy, young surgeon

## Abstract

**Background/Objectives**: Lumbar disc herniation, a complex challenge in spinal health, significantly impacts individuals across diverse age groups. This article delves into the intricacies of this condition, emphasising the pivotal role of anatomical considerations in its understanding and management. Additionally, lumbar discectomy might be considered an “easy” surgery; nevertheless, it carries significant risks. The aim of the study was to present a groundbreaking “three-step approach” with some anatomical insight derived from our comprehensive clinical experiences, designed to systematise the surgical approach and optimise the outcomes, especially for young spine surgeons. We highlighted the purpose of the study and introduced our research question(s) and the context surrounding them. **Methods**: This retrospective study involved patients treated for lumbar disc herniation at a single institution. The patient demographics, surgical details, and postoperative assessments were meticulously recorded. All surgeries were performed by a consistent surgical team. **Results**: A total of 847 patients of the 998 patients initially included completed the follow-up period. A three-step approach was performed for every patient. The recurrence rate was 1.89%. Furthermore, the incidence of lumbar instability and the need for reoperation were carefully examined, presenting a holistic view of the outcomes. **Conclusions**: The three-step approach emerged as a robust and effective strategy for addressing lumbar disc herniation. This structured approach ensures a safe and educational experience for young spinal surgeons.

## 1. Introduction

Lumbar disc herniations (LDHs) rank among the leading causes of lower back pain, impacting approximately 1%–3% of individuals annually, with a prevalence of about 12% [1]. It is noteworthy that a significant percentage (80%) of people will grapple with lower back discomfort at some stage in their lives, resulting in healthcare costs exceeding USD 100 billion per year in the US [2]. LDHs affect individuals of all ages and are directly correlated with increasing patient age [3]. This condition, characterised by the protrusion of intervertebral disc material, often leads to intense lower back pain and radiating symptoms, profoundly impacting the daily lives and prospects of those affected [2,4].

The gold-standard first-line treatment for an LDH is the conservative one, which includes oral medications, rest, and physical therapy; surgical interventions are reserved for cases unresponsive to conservative measures [5].

The evolution of discectomy techniques, from Mixter and Barrin in 1934, followed by Caspar and Williams in 1977, Wiltse and Spencerin and Kambin and Sampson in 1988, to Foley and Smith in 1997, marks significant progress in spinal surgery. Discectomy involves a small back incision to remove extruded disk material, relieving nerve pressure. While the primary goal of alleviating nerve impingement remains unchanged, newer methods focus on minimising trauma to the muscle and enhancing visualisation [5,6].

In this study, we present a three-step microsurgical approach with some important anatomical considerations that provide a straightforward and intuitive educational resource offering a streamlined and standardised surgical process.

Understanding the underlying anatomical factors, patient characteristics, and surgical techniques is important to optimise patient outcomes and reduce intraoperative and postoperative complications, guiding young spinal surgeons in the management of this prevalent pathology and integrating both theoretical knowledge and practical skills.

In this study, we present a three-step surgical approach with some important anatomical considerations that provide a straightforward and intuitive educational resource offering a streamlined and standardised surgical process.

## 2. Materials and Methods

### 2.1. Study Design

We conducted a retrospective study involving patients who underwent surgical treatment for lumbar disc herniation at the Mater Olbia Hospital between July 2019 and October 2022. The inclusion criteria for this study required patients to be 18 years old, diagnosed with lumbosacral disc herniation, and experiencing symptoms that were unresponsive to conservative medical therapy for a minimum of 6 weeks. A minimum follow-up period of 12 months was required. Patients with intraforaminal and extraforaminal disc herniation were not included in this study.

### 2.2. Data Collection

Patient data were collected before and after the surgical intervention to assess variables such as lower back pain, leg pain, quality of life, and psychosomatic aspects. Additional data included patient demographics such as age, weight, body mass index, smoking status, and other risk factors.

All surgeries were performed by a consistent team of two surgeons, ensuring uniformity in the surgical approach. The same surgical technique was employed for all patients.

### 2.3. Follow-Up

Patients underwent a one-month follow-up evaluation, which included lumbosacral X-rays. Annual follow-up assessments were conducted thereafter, resulting in a cumulative follow-up period of about 1 to 4 years for each patient.

### 2.4. Additional Data Collected

Intraoperative times were documented to assess the duration of surgery for each patient. Average hospital stay durations were recorded to understand the postoperative recovery times. Intraoperative and postoperative complications were documented and analysed. Recurrence rates of lumbar disc herniation and risk of subsequent steno-instability were evaluated during the follow-up period. In instances of recurrent lumbar or radicular pain, a comprehensive radiological reassessment was conducted, incorporating lumbar magnetic resonance imaging (MRI) and dynamic/standing plain radiographs. Recurrence, in this context, was characterised by the emergence of a fresh extrusion of the nucleus pulposus or an extruded fragment through the annulus, resulting in the constriction of the dural sac and impingement on the nerve root at the same precedent level [7]. Lumbar instability was delineated by the novel onset of spondylolisthesis or the degeneration of the facet joints. The radiological criteria outlined by Wang et al. were employed for an accurate evaluation [8].

All patients signed a written informed consent form, and the study was previously approved by the local ethics committee, protocol number 276/2020/CE. The data were analysed using appropriate statistical methods to assess the outcomes and implications of the surgical treatment of lumbar disc herniation in the specified patient population.

## 3. Surgical Technique

### 3.1. Anatomical Consideration

The lumbar segment of the vertebral column is an anatomical region notable for its role in weight-bearing, trunk mobility, and the transmission of neural signals to the lower extremities. Comprising five lumbar vertebrae (L1–L5) and the initial sacral vertebra (S1), the lumbar region stands out for its robust and load-bearing characteristics. The lumbar vertebrae exhibit wide vertebral bodies, the pedicles where they originate the transverse processes, the superior and inferior articulate facets that impart both flexibility and structural integrity and the two laminae that continue posteriorly into the spinous process [9,10].

Intervertebral discs, positioned between adjacent lumbar vertebrae, comprise a gel-like nucleus pulposus encased within a fibrous annulus fibrosus that act as shock absorbers and facilitate spinal movement. These discs are notably exposed to substantial biomechanical stresses, rendering them susceptible to degenerative changes and herniation, commonly associated with lumbar disc pathologies [11,12,13,14,15].

Lumbosacral spinal roots, emerging through intervertebral foramina in the lumbar region, perform the role of transmitting motor and sensory signals to the lower limbs [10,11].

Several spinal ligaments further enhance the stability and integrity of the lumbar region, including the anterior and posterior longitudinal ligaments, the ligamentum flavum, and the intra and supra-spinous ligaments [12].

### 3.2. Posterior Lumbar Surgical Triangle

**Figure 1:**
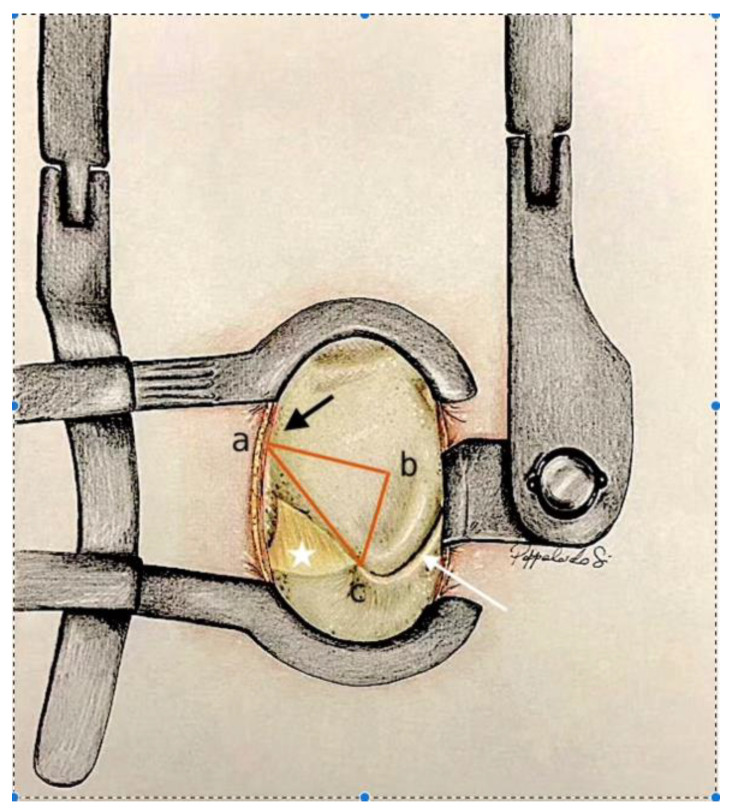
After the Caspar distractor position and skeletonisation of the homolateral spinous process and homolateral lamina, and the identification of the anatomical triangle: (**a**) midpoint on the base of the spinous process; (**b**) inferior third of the later portion of the lamina; (**c**) medial aspect of the inferior articular process. Yellow ligament (white star). The base of the spinous process (black arrow). Superior and inferior facets of the inferior articular process (white arrow).

The posterior lumbar anatomical triangle (Figure 1) is a pivotal anatomical region within the context of the lumbar spine, characterised by three distinct reference points:

- The midpoint of the base of the spinous process: the midline is defined by the alignment of the spinous processes of the lumbar vertebrae. The midsection of the base of each spinous process constitutes the first reference point within the triangle;
- The medial portion of the homolateral inferior articular process: the second reference point is the medial part of the inferior articular process of the same vertebra on the homolateral side;
- The inferior third portion of the homolateral lamina: the third reference point is the inferior third part of the lateral portion of the lamina of the same vertebra on the homolateral side.

This anatomical triangle holds great clinical relevance in spinal surgery. When the yellow ligament situated inferiorly is removed, it provides a clear and direct view of the medially located dural sac with the corresponding nerve root [11,12]. Furthermore, this visualisation enables the identification of the intervertebral disc, including any herniation that may protrude laterally and inferiorly, potentially compressing the nerve root.

### 3.3. Preoperative Evaluations

In the surgical setting, it is essential to adhere to established procedural norms to avert errors in patient selection, surgical level, or side [16]. Employing preoperative antibiotics is considered the gold standard, as they have unequivocally shown efficacy in diminishing postoperative infections [17]. While the use of elastic stockings and compressive boots is advised to mitigate venous thromboembolism, their definitive benefits are yet to be conclusively proven.

Typically, a urinary catheter is deemed unnecessary due to the anticipated brief duration of the surgical procedure. Intraoperative neuromonitoring is also generally not necessary.

If the patient’s condition permits, opting for general anaesthesia is advisable. This allows for the application of short-acting neuromuscular blocking agents, facilitating smoother muscular dissection and exposure. However, it is crucial that these agents have dissipated or have been reversed before decompression. When operating around the nerve root, the heat generated from cautery or compression during bone work can provoke the stimulation of the root. This stimulation serves as a valuable warning sign for the surgeon, indicating excessive stress on the nerve. Furthermore, the mobility permitted using local anaesthetics poses a potential risk when maintaining fixed retraction or operating in proximity to neural elements [4,16].

### 3.4. Positioning

The effectiveness of exposure in lumbar discectomy is directly impacted by the alignment of neighbouring vertebrae and indirectly affected by the extent of intraoperative bleeding. Consequently, the objective in positioning for lumbar microdiscectomy is to enhance lumbosacral flexion and facilitate smoother access through the interlaminar space.

**Figure 2:**
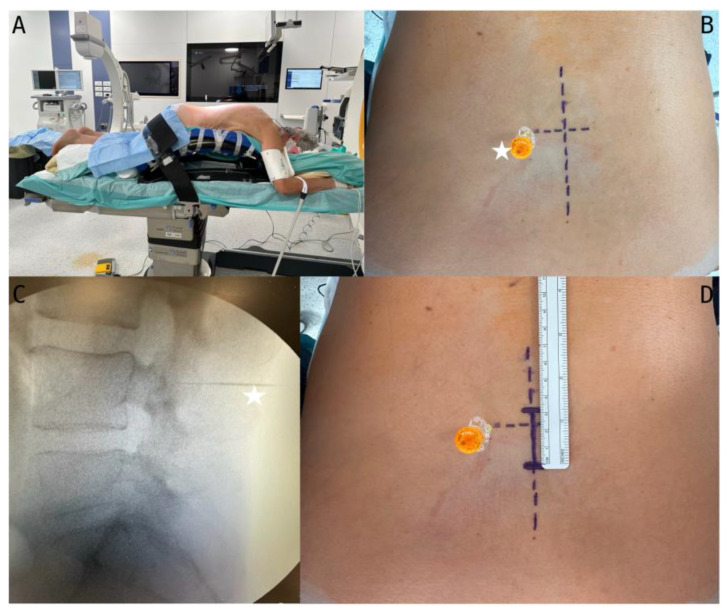
(**A**) Positioning on a Wilson frame and surgical room setting; (**B**) spinal needle (white star) positioned laterally on the contralateral articular process of the intended surgical site; the long line corresponds to the midline, and the short line corresponds to the level for L4L5; (**C**) X-ray control of the correct L4 level; (**D**) drawn incision extending one-third superior and two-thirds inferiorly to the point level of about 3 cm.

Positioning the patient in a prone manner involves placing them on a Wilson frame or Kambin frame, ensuring that the surgical area aligns with the apex of the frame. Minimising pressure on the abdomen is crucial to reduce airway pressures, epidural venous congestion, and intrathecal pressure. The patient’s arms should be slightly abducted and extended in a cephalad direction, resembling the “Superman” position (Figure 2A). This positioning provides the surgeon with increased working space and facilitates X-ray or fluoroscopy when obtaining a localisation [18,19,20,21].

To prevent brachial plexus injury, shoulder extension and abduction should be limited to less than 90 degrees.

All pressure points must be adequately padded to prevent pressure-related ischemia, with particular attention to protecting the ulnar nerve by padding the medial condyle of the elbow.

### 3.5. Surgical Level Localisation

Following patient positioning, one should thoroughly disinfect the operative field. The intercristal line is used to identify the space between the fourth and fifth lumbar vertebrae. Subsequently, pinpointing the specific lumbar level, one should insert an 18-gauge spinal needle laterally on the contralateral articular process of the intended surgical site (Figure 2B,D). An X-ray is then performed to confirm the location of the needle before marking the skin incision (Figure 2C). This technique ensures an accurate incision starting point, minimises the risk of infectious agent transfer and bleeding, and reduces the chance of impacting the dural sac and nerve root due to the lateral needle placement.

## 4. Three-Step Approach for Lumbar Disk Herniation

### 4.1. Step One: Exposure and Skeletal Visualisation

**Figure 3:**
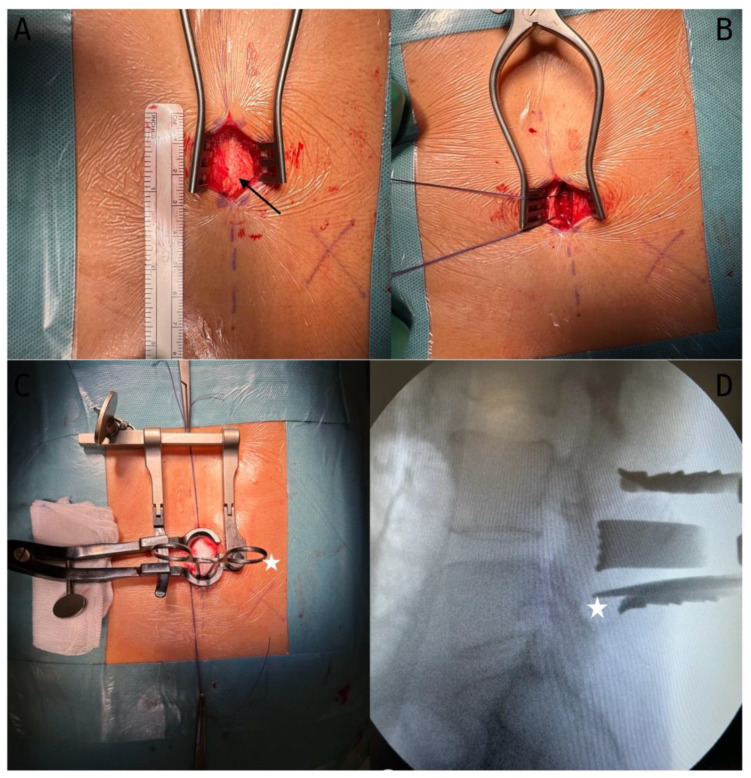
(**A**) Incision and muscular fascia (black arrow) exposition; (**B**) muscle fascia handling; (**C**) skeletonisation of the homolateral lamina and placement of a Caspar distractor. Klemmer forceps are positioned under the lamina for X-ray control (white star); (**D**) X-ray control of the correct Caspar positioning on the right level.

A midline cutaneous incision of approximately 2.5–3 cm is made, ensuring a focused and minimalistic approach. The paramedian curvilinear incision of the muscle fascia on the symptomatic side allows targeted access to the affected area (Figure 3A).

The muscle fascia on the symptomatic side is delicately dissected, preserving muscle integrity while gaining optimal exposure. The suspension of the dissected fascia using a silk thread provides a controlled and clear view of the surgical field (Figure 3B).

The skeletonisation of the hemilamina of the upper vertebra is performed to access the targeted vertebral structures.

At this point, a Caspar or Scoville distractor is placed to facilitate controlled separation and visualisation (Figure 3C) and a radioscopic confirmation of the correct vertebral level is conducted by positioning a Penfield dissector or Klemmer forceps on the lower edge of the lamina, ensuring accuracy and alignment throughout the procedure (Figure 3D). This step is crucial for maintaining the integrity of the adjacent structures and guiding subsequent surgical actions.

### 4.2. Step Two: Microscopic Identification and Decompression

With the guidance of an intraoperative microscope, the surgical field is magnified to facilitate visualisation. Utilising the previously described anatomical triangle, the surgical team locates the midline (the base of the spinous process), medial portion of the inferior articular process, and lateral part of the inferior third portion of the lamina (Figure 1).

A high-speed drill with a diamond-tipped burr is employed for the careful removal of the inferior third of the hemilamina and the medial one-third of the inferior articular facet. The cephalad insertion of the yellow ligament is detached with a Kerrison rongeur that can be used as a curette or to cut the bone. If the drilling is correctly performed, thanks to the recognition of the anatomical landmarks, one bite is usually sufficient to see the epidural fat emerging beyond the end of the yellow ligament.

**Figure 4:**
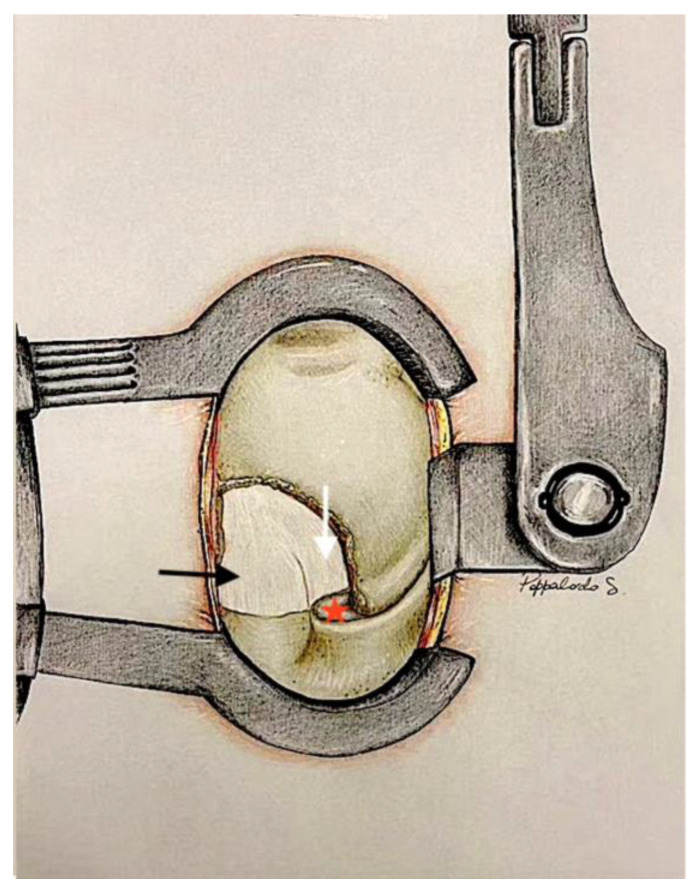
Exposition after the inferior third of the lamina, inferior articular facet, and yellow ligament removal (STEP 2). Removing one-third of the medial aspect of the inferior articular process and the inferior third of the homolateral lamina is necessary and sufficient to expose the dural sac (black arrow) and the nerve root (white arrow). The articular facet of the superior articular process (red star).

The yellow ligament is then delicately lifted and removed by placing the Kerrison forceps under the lower part of the vertebral lamina at the midline, where the dura is less attached. The forceps move in a super-caudal direction, then are rotated inferno-caudally to gently detach the ligament from the base of the spinous process. Subsequently, using the Weil forceps, the ligament is carefully removed, shifting it laterally toward the articular process under the direct visualisation of the epidural fat, identifying the dural sac and the nerve root compressed by the herniated disc (Figure 4).

### 4.3. Step Three: Hernia Exposure, Herniotomy and Discectomy, and Closure

**Figure 5:**
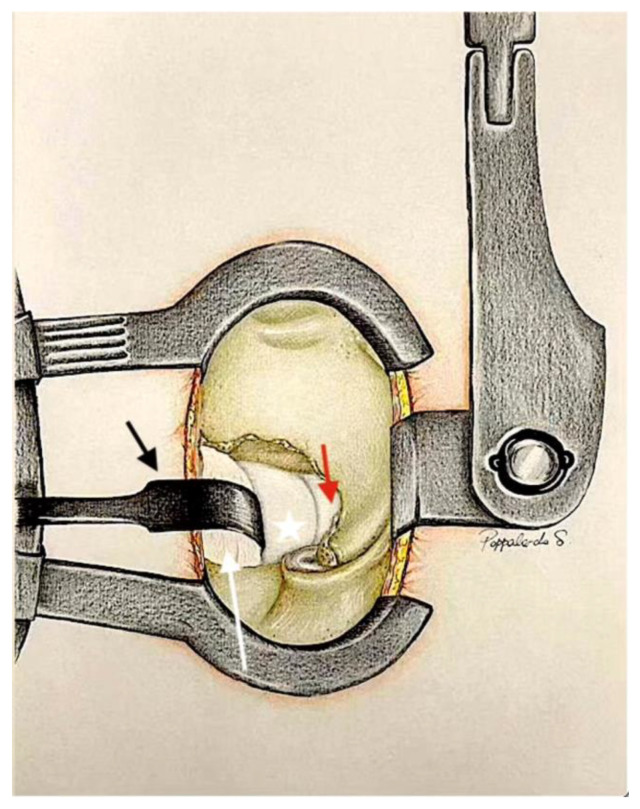
Hernia (white star) exposure after dural sac and nerve root retraction (white arrow). The third surgical step continues with the incision of the herniated ligament and intervertebral disc and the asportation of the hernia with its residual fragments. Retractor (black arrow); intervertebral disk (red arrow).

Using a dural dissector, a Brunner/Meyerding retractor, or a spatula, a gentle medial retraction is applied to the dural sac and the nerve root, exposing the herniated disc (Figure 5).

Only after the identification of the nerve root, if necessary, bipolar coagulation may be used to prevent bleeding; then an incision, parallel to the dural edge, is made into the ligament containing the herniated disc, providing access for removal. The delicate removal of the herniated disc is carried out using Weil or Caspar forceps, ensuring precision and minimal disruption to the surrounding structures.

A hooked dissector is used to carefully examine for any residual herniated fragments beneath the nerve root. The foraminotomy of the involved nerve root is performed, if necessary, to ensure complete decompression.

Thorough haemostasis is achieved to prevent bleeding complications.

The muscle fascia is meticulously closed, ensuring optimal structural integrity. Subcutaneous skin closure is performed with attention to cosmesis.

## 5. Results

During the period between July 2019 and October 2022, 998 patients underwent surgery for lumbar disc herniation at Mater Olbia Hospital. One hundred and fifty-one patients were excluded from the present study because of a follow-up duration of less than one year. As a consequence, 847 patients were included. The patient cohort, comprising 487 males and 360 females, exhibited a mean age of 51.42 (±13.4) years (Table 1).

Operatively, a total of 866 lumbar hernias were addressed, with 828 occurring at a single level and 19 at two different levels. Herniations were observed at the L5S1 level (345 cases), L4L5 level (370 cases), L3L4 level (107 cases), L2L3 level (42 cases), and L1L2 level (2 cases), showcasing the frequency distribution across the lumbar levels (Table 2).

During the surgical observations, both through the physical measurements and intraoperative microscope images (Microscope Leica M530 OHX, Leica Microsystems), we established the dimensions of the three sides of the posterior lumbar anatomical triangle.

The first side (from the base of the spinous process to the lateral point of the third inferior part of the lamina) exhibited an average length of 18.1 (±3.82) mm. Moving to the second side (from the lateral point of the third inferior part of the lamina to the inferior articular facet), the average length measured 14.6 (±0.85) mm. On the third side (from the inferior articular facet to the base of the spinous process), the average length was 20.4 (±2.55) mm. Moreover, the area of bone removed from the lamina, approximately corresponding to the triangular area, was measured. The average area of this triangle, representing the removed bone, was 164.87 (±37.9) mm^2^ (Table 3).

The closure procedures involved the use of an absorbable suture (Vicryl Rapid 2-0). Drainage was never used. The average surgical time was 52 min, with a standard deviation of 16 min. The average hospital stay was 2.40 (±0.65) days (Table 2).

Intraoperative complications included seventeen cases of cerebrospinal fluid leaks, one vertebral fracture, one subcutaneous hematoma, and one instance of sensory deficit (Table 4). Wound management proved successful, with an average wound size of 3.28 cm and no postoperative infectious complications reported (Table 2).

The functional and pain-related outcomes demonstrated substantial improvements. The preoperative measures revealed a mean Oswestry disability index of 52.43% (±20.61), which significantly decreased to 21.43% (±14.92) postoperatively. Similarly, the preoperative VAS scores for leg (7.76 ± 1.64) and back pain (7.76 ± 1.61) were substantially reduced postoperatively, which were, respectively, 1.60 ± 1.70 and 2.37 ± 1.92. The Euro quality of life 5 dimensions preoperative mean was 0.383 (±0.21), increasing to 0.697 (±0.21) postoperatively (Table 5).

We identified a symptomatic recurrent disc herniation not responsive to medical treatment in 16 cases, representing an incidence rate of 1.84%. These individuals underwent a secondary surgical procedure involving the removal of the recurrence and a new discectomy. Importantly, all 16 patients experienced complete resolution of clinical symptoms following the additional intervention.

Simultaneously, we observed a risk of instability necessitating posterior lumbar fusion with rods and screws in 19 cases, constituting an incidence rate of 2.19% (Table 4).

## 6. Discussion

The three-step approach for lumbar disc herniation, characterised by meticulous exposure, decompression, and closure techniques, yields favourable surgical outcomes. The precision-oriented steps contribute to enhanced patient recovery and long-term well-being.

In our investigation, we encountered a recurrence rate of 1.84%, a lumbar instability rate of 2.19%, a dural tear incidence of 2%, and a reintervention rate of 4.03%. Importantly, no instances of superficial or deep infections were recorded. Subcutaneous hematoma and sensory deficit were present only in one patient, respectively. These findings present favourable statistics when compared to the existing literature. Such promising results could be attributed to the nuanced anatomical and surgical considerations elucidated in our study.

In our case series, 16 patients (1.84%) underwent surgery for recurrent disc herniation. This result is in line with previous literature that has identified recurrent lumbar disc herniation as a common complication after discectomy, with reported frequencies of up to 21%. Identifying risk factors for recurrence remains controversial; clinical factors like age, smoking, gender, and obesity have been explored, but findings vary across studies [22,23,24,25]. Radiological considerations, such as disc degeneration, increased disc height, and larger sagittal range of motion in flexion–extension radiography, are also implicated [26,27,28,29,30]. Mc Girt et al. reported the incidence of recurrent disc herniation in 21 limited discectomy cohorts (n = 5832 patients) and in 25 aggressive discectomy cohorts (n = 6114 patients). The mean reported incidence of recurrent disc herniation after limited discectomy was 7%. The reported incidence ranged from 2 to 18%. The mean reported incidence of recurrent disc herniation after aggressive discectomy was 3.5%. The reported incidence ranged from 0 to 9.5% [31].

Here, we reported an intraoperative dural tear rate of 1.96%, with no case of postoperative CSF leak, as documented in other studies regarding primary discectomy, which have reported an incidence rate between 1.3% and 3.5% [32,33,34,35].

Instances of wound complications, including superficial or deep infections, are infrequent after lumbar discectomy. According to Pugely et al., the overall occurrence of wound complications was reported at 1.88% in the inpatient setting and 1.21% in the outpatient setting [36]. Among 7464 patients examined in the NSQIP database, Esfahani et al. observed an overall wound complication rate of 1.1%. In terms of infections, neurosurgeons had rates of 0.6% for superficial infections and 0.2% for deep infections [37]. Shriver et al. found wound complication rates of 2.1% and 1.2% for open discectomy [38]. Harper et al. found an incidence of 2.2%, and twenty-nine patients (82.9%) were treated with operative debridement [39]. No infections were reported in the present study. This could be attributed to the surgical technique or, more probably, it was just by chance because we are aware that zero-infection surgery is probably impossible to achieve.

Heindel et al. reported that lumbar spinal fusion was performed on 5.9% (370/6274) of patients within 4 years, and patients who received a re-exploration discectomy within 2 years of the index procedure went on to receive lumbar fusion at a rate of 38.4% (48/125) within the 4 years after the re-exploration discectomy [40]. Österman et al. reported a rate of 2.8% in a population of 35,309 patients who underwent an initial lumbar discectomy in the Finnish hospital discharge register over the time span of 11 years [41]. Moreover, Heindel et al. reported a lumbar fusion rate of 5.9% four years after initial lumbar discectomy within 13,654 patients in the Humana database [40]. Castillo et al. found a 4.97% rate of fusion four years after initial lumbar discectomy within a population of 68,305 patients from the Truven Healthcare Analytics Marketscan Research Database (Marketscan) [42]. We found similar results in our cohort.

In a meta-analysis conducted by Bombieri et al., various discectomy approaches from 1997 to 2020 were evaluated, including open, microlumbar, microendoscopic, and fully endoscopic techniques [43]. Comparing our results with the other approaches, our recurrence rate of 1.84% is lower than all the reported techniques, including open discectomy (4.1%), microlumbar discectomy (5.1%), microendoscopic discectomy (3.9%), and fully endoscopic discectomy (3.5%). Our reoperation rate of 4.03% is also lower compared to open discectomy (5.2%), microlumbar discectomy (7.5%), and microendoscopic discectomy (4.9%), and slightly lower than fully endoscopic discectomy (4%). The dural tear rate in our approach (1.96%) is significantly lower than in open discectomy (6.6%) and microendoscopic discectomy (4.4%) and is comparable to microlumbar discectomy (2.3%) and fully endoscopic discectomy (1.1%). Our wound infection rate is significantly lower than all other techniques reported: open discectomy (3.5%), microlumbar discectomy (7.5%), microendoscopic discectomy (4.9%), and fully endoscopic discectomy (4%). These findings suggest that our three-step approach to microlumbar discectomy offers superior outcomes when compared to the various techniques reviewed in the meta-analysis, highlighting its potential to improve patient outcomes and minimise postoperative complications.

The controlled exposure of the three-step approach ensures a clear and targeted view of the herniated disc and the surrounding structures; minimised tissue trauma and precise anatomical visualisation contribute to reduced postoperative discomfort. The removal of the herniated disc with delicacy and attention to the anatomical triangle ensures the thorough decompression of the spinal cord and nerve root, and the systematic examination for residual herniated fragments under the nerve root, coupled with foraminotomy, minimises the chances of postoperative complications and ensures the likelihood of sustained symptom relief and functional improvement over the long term. This approach facilitates a quicker recovery timeline and contributes to shorter hospital stays and a faster return to daily activities. The meticulous identification of the anatomical triangle and the systematic adherence to the three surgical steps can serve as a robust foundation and a valuable exercise, especially for residents or young spinal surgeons, providing a secure and educational framework for addressing disc herniation.

The standardisation and simplification of the approach in a step-by-step procedure allow educators to safely guide young surgeons during the procedure because the more experienced one can easily take control of the surgery at any moment without additional risk for the patient or unnecessary waste of time. Each phase of the approach is thought to be the safest possible, even if a trainee is involved; the lateral needle placement, for example, but also the use of high-speed drilling only in a limited fashion, where the dura is still covered by the yellow ligament; the use of microscope magnification and the care taken to the identification of the nerve root before coagulation or disc incision. As for every surgery, even lumbar discectomy should always be refined to improve the risk–benefit balance, also in an educational context.

## 7. Conclusions

In conclusion, the three-step approach proved effective in achieving favourable surgical outcomes for lumbar disc herniation. Moreover, it allows the education of young surgeons with limited risks.

## Figures and Tables

**Table 1:** Demographic and follow-up data.

Demographic Data	Number of Patients	Other Data	
Patients	847	Average age	51.42 years (±13.40)
Employed/non-employed	544/303	Male/female ratio	487/360
Smokers/non-smokers	281/566	Mean BMI	25.51 (±4.06)
Arterial hypertension	228	Mean follow-up	12–48 months
Previous lumbar surgery	107		
Fibromyalgia	54		

**Table 2:** Level and surgical data.

Lumbar Hernia Treated	Number of Levels	Surgical Data	
Total lumbar hernia	866	Average wound size	3.28 cm (±0.67)
Single level	828	Mean surgical time	52 min (±16)
Double level	19	Average hospital stay	2.40 days (±0.65)
L5S1	345	Average wound size	3.28 cm (±0.67)
L4L5	370		
L3L4	107		
L2L3	42		
L2L1	2		

**Table 3:** Dimension of the bone landmark in the surgical field.

Surgical Triangle Dimension	Characteristics/Borders	Average Length (mm)	Standard Deviation
First side	From the base of the spinous process to the lateral point of the third inferior part of the lamina	18.1	3.82
Second side	From the lateral point of the third inferior part of the lamina to the inferior articular facet	14.6	0.85
Third side	From the inferior articular facet to the base of the spinous process	20.4	2.55
Area of drilled bone	Triangular area	164.87 mm^2^	37.9

**Table 4:** Complications.

Intraoperative Complications	Number of Complications	%	Postoperative Complications	Number of Complications	%
Dural tear	17	1.96	Recurrence	16	1.84
Vertebral fracture	1	0.11	Instability	19	2.19
Subcutaneous hematoma	1	0.11	Motor deficit	0	0
Dural tear	17	1.96	Sensory deficit	1	0.11
			Wound infection	0	0

**Table 5:** Preoperative and postoperative patient assessments.

Patient Assessment	Preoperative	Postoperative
ODI (Oswestry disability index)	52.43% (±20.61)	21.43% (±14.92)
VAS (visual analogue scale) leg pain	8.05 (±1.64)	1.60 (±1.70)
VAS (visual analogue scale) back pain	7.76 (±1.61)	2.37 (±1.92)
EQ-5D (Euro quality of life 5 dimensions)	0.383 (±0.21)	0.697 (±0.21)

## Data Availability

The data presented in this study are available on request from the corresponding author.
